# The Practice of Medicine

**Published:** 1902-03

**Authors:** 


The Practice of Medicine. A Text-Book for Practitioners and Students, with Special Reference to Diagnosis and Treatment. By James Tyson, M. D., Professor of Medicine in the University of Pennsylvania, and Physician to the Hospital of the University; Physician to the Philadelphia Hospital; Fellow of the College of Physicians of Philadelphia; Member of the Association of American Physicians, etc. Second edition. Thoroughly revised, and in parts rewritten. With 127 illustrations, including colored plates. 1222 pages. Price, cloth, $5.50. P. Blakiston's Son & Co., Publishers, 1012 Walnut St., Philadelphia.

No text-book on medical practice issued in recent years has been received with more favor by the profession at large than the first edition of Prof. Tyson's Practice. The book became a standard one with medical colleges and physicians almost from the first. It was written from the standpoint of an eminent medical practitioner and teacher, and is based largely on the author's personal practice. The book is intended to teach mainly diagnosis and treatment, the most essential and practical branches of medical science. It is an evidence of the special favor with which the first edition was received that a second one was demanded by the profession in less than four years after the publication of the first. This is very complimentary to the author, but those who are so fortunate as to secure a copy of this second edition are the real gainers.

In the preparation of this second edition, the author has made many additions, some corrections, and has introduced much new matter, bringing the work up to date, as the progress of medicine demanded. More changes have been made in the present edition in the sections on infectious diseases and diseases of the nervous systems, about three hundred pages being devoted to each of these sections. The book is divided into fifteen sections, as follows: I, Infectious Diseases; II, Diseases of the Digestive System; III, Diseases of the Respiratory System; IV, Diseases of the Heart and Blood-Vessels; V, Diseases of the Blood and Blood-Making Organs; VI, Diseases of the Thyroid Gland; VII, Diseases of the Urinary Organs; VIII, Diseases of the Suprarenal Capsule; IX, Constitutional Diseases; X, Diseases of the Nervous System; XI, Diseases of the Muscular System; XII, The Intoxications; XIII, Exposure to High though Bearable Temperature; XIV, Animal Parasites and the Conditions Caused by Them; XV, Summary of Symptoms Following Overdose of Poisons, and the Treatment of Their Effects. To which is added a table of minimum dose which has caused death, and maximum dose followed by recovery. The work also has an appendix giving tables for the conversion of the English into Metric system, and the reverse. Another point of superiority in this very valuable work is its most complete index, a matter too often overlooked by the authors of otherwise meritorious books.

